# Integrative transcriptomic analysis identifies CCL22-associated immune signatures in air pollution-related atopic dermatitis

**DOI:** 10.3389/fpubh.2026.1906298

**Published:** 2026-09-03

**Authors:** Chang Gao, Liping Chen, Tianfeng Huang, Zi Wang

**Affiliations:** 1 Faculty of Medical Instrumentation, Shanghai University of Medicine and Health Sciences, Shanghai, China; 2 Department of Anesthesiology, The First Affiliated Hospital of Wenzhou Medical University, Wenzhou, Zhejiang, China; 3 Yangzhou Key Laboratory of Anesthesiology, Northern Jiangsu People's Hospital Affiliated to Yangzhou University, Yangzhou, Jiangsu, China; 4 Yangzhou University Medical College, Yangzhou, Jiangsu, China; 5 Key Laboratory of Anesthesiology (Shanghai Jiao Tong University), Ministry of Education, Shanghai, China

**Keywords:** air pollution, atopic dermatitis, CCL22, environmental health, exposome, immune microenvironment, machine learning, transcriptomics

## Abstract

**Background:**

Air pollution has been associated with the development and exacerbation of atopic dermatitis (AD), but the molecular signatures connecting pollutant-related targets with AD-associated immune dysregulation remain incompletely characterized.

**Methods:**

We applied an integrated systems toxicology and transcriptomic framework to prioritize candidate pollutant-related immune signatures in AD. Pollutant-associated targets were intersected with high-confidence AD-related genes, followed by protein–protein interaction analysis, GO/KEGG enrichment, machine learning, immune infiltration analysis, single-cell transcriptomics, *in silico* CCL22 perturbation, 1-fluoro-2, 4-dinitrobenzene (DNFB)-induced AD-like mouse validation, and exploratory molecular docking.

**Results:**

Shared pollutant–AD targets were mainly enriched in cytokine activity, chemokine signaling, pattern-recognition receptor activity, IL-17 signaling, cytokine–cytokine receptor interaction, and Toll-like receptor-related inflammatory pathways. A machine learning framework based on 15 algorithms and 175 predictive combinations identified plsRglm + AdaBoost as the optimal model, with an AUC of 0.963 in the training cohort and AUCs of 1.000, 0.909, and 0.966 in three validation cohorts. The model identified a pollutant-prioritized AD signature including CCL22, CCL5, CSF2, F2RL1, HRH4, ICAM1, IFNG, IL10, IL17A, and IL18. CCL22 was upregulated in AD samples and mainly localized to dendritic cells and macrophages. *In silico* CCL22 perturbation was associated with extracellular matrix and stromal remodeling programs, while DNFB-induced AD-like dermatitis confirmed increased CCL22 expression in lesional skin.

**Conclusion:**

These findings identify CCL22-associated immune and stromal remodeling signatures as candidate molecular features of air pollution-related AD and generate testable hypotheses for future controlled exposure studies.

## 1 Introduction

Atopic dermatitis (AD) is a chronic, relapsing inflammatory skin disease characterized by intense pruritus, epidermal barrier dysfunction, and immune dysregulation (1). Although type 2 inflammation driven by interleukin (IL)-4 and IL-13 represents a central immunological feature of AD, increasing evidence indicates that AD is a heterogeneous disease involving multiple immune axes, including Th1-, Th17-, Th22-, innate immune-, and chemokine-mediated responses (1, 2). These immune abnormalities interact with impaired barrier integrity and environmental exposures, leading to recurrent skin inflammation, immune-cell infiltration, and disease exacerbation. This heterogeneity is increasingly conceptualized as distinct molecular endotypes; within this framework, CCL17/CCL22–CCR4 signaling represents a recognized type 2-associated feature rather than a pollution-specific pathway (2, 3).

Air pollution has emerged as an important environmental factor associated with the development and aggravation of AD. Epidemiological and mechanistic studies have linked exposure to particulate matter, nitrogen oxides, sulfur dioxide, ozone, carbon monoxide, traffic-related air pollutants, and volatile organic compounds (VOCs) with increased AD risk, disease activity, and healthcare utilization (4–6). A systematic review and meta-analysis of adult AD reported that outdoor air pollutants may exert both short-term and long-term adverse effects on AD development, severity, and symptom exacerbation (7). Mechanistically, air pollutants can induce oxidative stress, disrupt epidermal barrier proteins, alter transepidermal water loss, and activate inflammatory signaling pathways in skin tissues (4, 8). In particular, PM2.5 exposure has been shown to reduce filaggrin and other barrier-related proteins through TNF-α- and aryl hydrocarbon receptor-dependent mechanisms, thereby compromising skin barrier function (8). In addition, recent evidence suggests that VOCs, including toluene, may be associated with AD pathogenesis in adults (9).

Despite these epidemiological and experimental observations, the molecular associations linking air pollutant-related targets with AD-associated immune dysregulation remain incompletely understood. Conventional toxicological studies often focus on single pollutants or individual molecular targets, whereas real-world air pollution exposure usually consists of complex mixtures that may converge on shared inflammatory pathways. Network toxicology provides a systems-level approach to integrate pollutant-associated genes, disease-related targets, protein–protein interaction networks, and pathway enrichment analyses, thereby helping to identify candidate molecular relationships between environmental exposure and disease phenotypes (10, 11). When combined with transcriptomic machine learning, single-cell analysis, and molecular docking, this strategy may provide a more comprehensive framework for identifying pollutant-informed immune biomarkers and testable candidate targets.

In this study, we hypothesized that pollutant-associated targets may converge with inflammatory and chemokine-related programs in AD. We first identified overlapping genes between air pollutant-associated targets and AD-related genes, followed by protein–protein interaction and functional enrichment analyses. We then applied machine learning across transcriptomic datasets to construct and validate a pollutant-prioritized diagnostic gene signature for AD. Single-cell transcriptomic analysis was used to characterize the cellular localization and network-level relevance of the candidate gene CCL22. A DNFB-induced AD-like dermatitis mouse model was used only to assess whether CCL22 is upregulated in inflammatory skin lesions and was not intended to model pollutant exposure. Finally, exploratory molecular docking was performed to generate structural hypotheses for selected pollutant–target pairs.

## 2 Materials and methods

### 2.1 Study design

**Figure 1:**
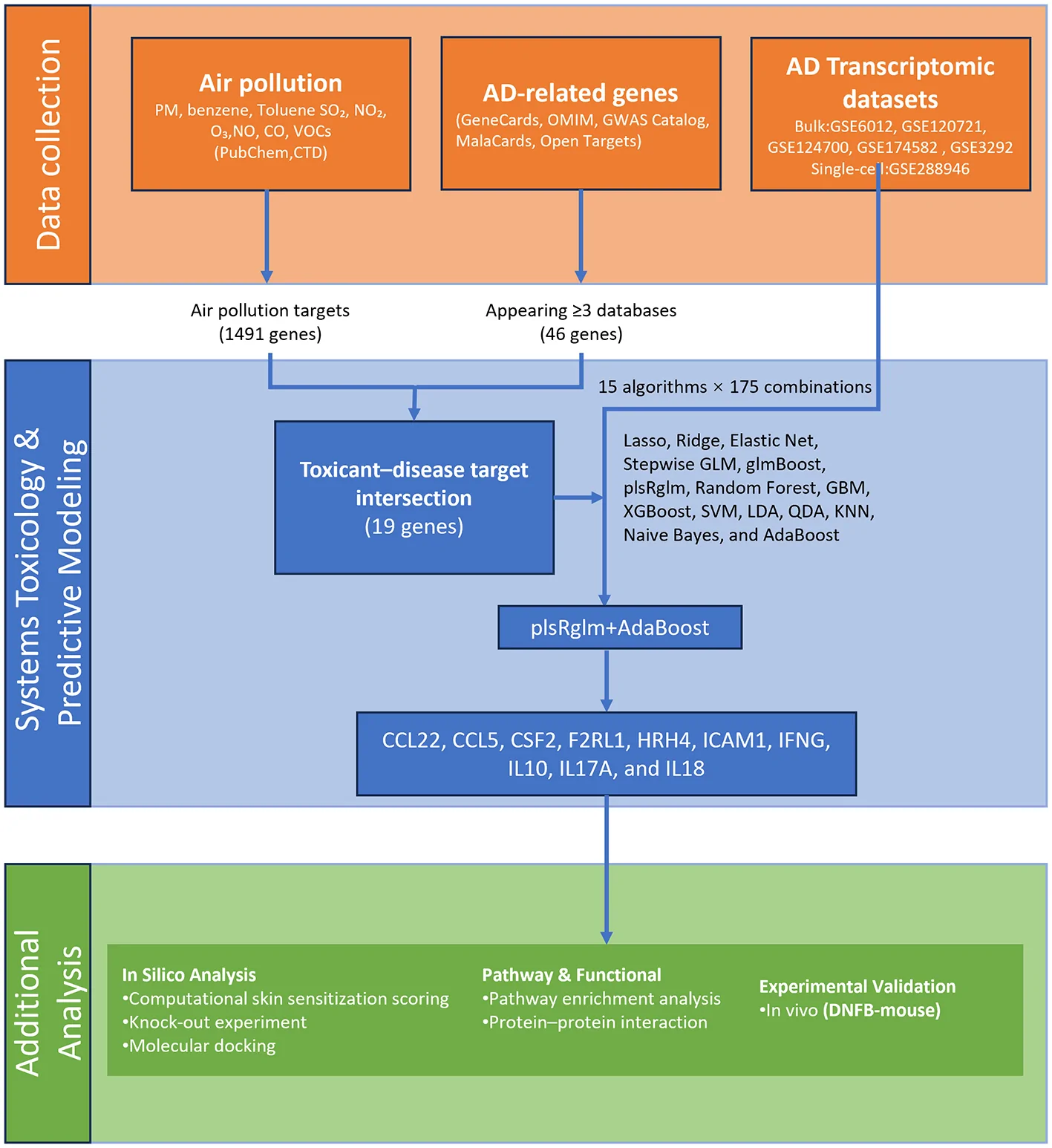
Overview of the study design and analytical framework. PM, particulate matter; SO_2_, sulfur dioxide; NO_2_, nitrogen dioxide; O_3_, ozone; NO, nitric oxide; CO, carbon monoxide; VOCs, volatile organic compounds; CTD, Comparative Toxicogenomics Database; OMIM, Online Mendelian Inheritance in Man; GWAS, genomewide association study; GEO, Gene Expression Omnibus; Lasso, least absolute shrinkage and selection operator; Ridge, ridge regression; Enet, elastic net; Stepglm, stepwise generalized linear model; XGBoost, extreme gradient boosting; RF, random forest; GBM, generalized boosted regression modeling; plsRglm, partial least squares regression for generalized linear models; LDA, linear discriminant analysis; SVM, support vector machine; PPI, protein–protein interaction.

This study was designed to characterize candidate molecular associations between air pollution-related targets and AD using an integrated systems toxicology framework. First, air pollutant-associated genes and AD-related genes were collected from public databases, and their intersection was used to prioritize shared candidate targets. Protein–protein interaction network construction and functional enrichment analyses were performed to characterize the biological functions and pathways represented by these targets. Machine learning models were then constructed using transcriptomic datasets to identify a pollutant-prioritized diagnostic gene signature for AD. Single-cell transcriptomic analysis was used to determine the cellular localization and predicted network-level relevance of CCL22. The computational findings were further assessed in a DNFB-induced AD-like dermatitis mouse model by measuring CCL22 protein expression, Ccl22 mRNA expression, and CCL22 immunohistochemical staining in lesional skin. Finally, exploratory molecular docking was performed to predict potential low-energy poses between structurally defined air pollutants and AD-related immune targets (Figure 1).

### 2.2 Data sources

Transcriptomic datasets used in this study were obtained from the Gene Expression Omnibus (GEO) database. Five bulk AD transcriptomic datasets based on human skin biopsy samples were included: GSE6012 (12), GSE120721 (13) were used as training cohorts, whereas GSE124700 (14), GSE174582 (15), GSE32924 (16) were used as independent validation cohorts. These datasets included lesional AD skin, nonlesional AD skin, and healthy control skin samples, depending on the original study design. Dataset information, including GEO accession numbers, sample sources, disease/control composition, and platform details, is summarized in Supplementary Table S1. Raw expression matrices were processed independently for each dataset. Gene expression values were log_2_-transformed when necessary and normalized within each cohort. Batch effects in the training datasets were adjusted using the **sva** R package before model construction. To avoid cross-study technic al confounding, validation datasets were processed independently and used only for external model evaluation. Datasets were eligible if they profiled human skin biopsies, included clearly annotated AD and healthy-control samples, and provided accessible expression matrices and phenotype data; non-skin datasets, datasets without controls, and duplicate cohorts were excluded. Samples without unambiguous disease-group annotation were not used, and unavailable platform-level gene measurements were not imputed. Expression matrices were processed independently using platform-appropriate normalization, and batch correction was restricted to the merged training cohorts. Across platforms, probe or transcript identifiers were mapped to official human gene symbols using the corresponding platform annotations, and duplicate identifiers mapping to the same gene were collapsed. Because processed matrices from different technologies were analyzed, raw data were not realigned to a single reference-genome build. Age and sex were not consistently available across all cohorts and therefore could not be included as harmonized covariates.

Single-cell RNA sequencing data were obtained from GSE288946. AD and healthy control skin samples were analyzed using the **Seurat** R package. Low-quality cells were removed according to standard quality-control metrics, including the number of detected genes, total RNA counts, and mitochondrial gene percentage. Data normalization, variable feature selection, dimensionality reduction, clustering, and cell-type annotation were performed using the standard Seurat workflow. Batch correction was conducted using the Harmony algorithm when required. The processed single-cell expression matrix was used for cell-type localization of candidate genes and subsequent *in silico* CCL22 perturbation analysis.

### 2.3 Literature search and study selection

To provide epidemiological context, PubMed and the Web of Science Core Collection were searched from database inception to June 30, 2026. The search combined disease-related terms (‘atopic dermatitis' OR ‘atopic eczema' OR ‘eczema') with exposure-related terms (‘air pollution' OR ‘particulate matter' OR ‘PM2.5' OR ‘PM10' OR ‘nitrogen dioxide' OR ‘NO_2_' OR ‘sulfur dioxide' OR ‘SO_2_' OR ‘ozone' OR ‘carbon monoxide' OR ‘volatile organic compounds' OR ‘traffic-related air pollution' OR ‘wildfire smoke'). Eligible studies were human observational studies evaluating AD incidence, prevalence, disease activity, symptoms, flares, or health-care utilization in relation to measured, modeled, or residence-linked air pollutant exposure. Reviews, editorials, case reports, conference abstracts without sufficient data, animal or *in vitro* studies, studies without AD-specific outcomes, and duplicate reports were excluded. Reference lists of relevant reviews were additionally screened. This structured literature search was used for narrative epidemiological context and was not registered as a standalone systematic review or used for quantitative meta-analysis.

### 2.4 Identification and characterization of air pollutants

To explore the potential immunotoxic effects of air pollutants relevant to AD, nine representative airborne pollutants were selected based on environmental prevalence and biological relevance: particulate matter (PM), benzene, sulfur dioxide (SO_2_), nitrogen dioxide (NO_2_), ozone (O_3_), nitric oxide (NO), carbon monoxide (CO), toluene, and volatile organic compounds (VOCs). These pollutants were used to retrieve associated human target genes from toxicogenomic databases.

For analyses requiring defined molecular structures, including *in silico* toxicity prediction and molecular docking, seven structurally defined pollutants were included: benzene, SO_2_, NO_2_, O_3_, NO, CO, and toluene. PM and VOC mixtures were excluded from molecular docking because they do not represent single compounds with stable and well-defined three-dimensional structures compatible with standard docking workflows. However, these pollutants were retained in the epidemiological, toxicogenomic, and transcriptomic analyses.

The SMILES notations and physicochemical properties of structurally defined pollutants were obtained from the PubChem database. Toxicological descriptors, including skin sensitization potential, SR-ARE activity, and SR-MMP activity, were predicted using ADMETlab 3.0. SR-ARE reflects oxidative stress response element activation, whereas SR-MMP reflects matrix metalloproteinase-related toxicity. Pollutants with a predicted skin sensitization score ≥0.5 were considered potential skin sensitizers.

### 2.5 Prediction of pollutant-associated human target genes

To identify potential human targets affected by air pollutants, chemical–gene interaction data were retrieved from the Comparative Toxicogenomics Database (CTD). For each pollutant, including PM, benzene, SO_2_, NO_2_, O_3_, NO, CO, toluene, and VOCs, human gene targets were extracted based on curated chemical–gene interaction records. Only genes supported by direct or inferred evidence in humans were retained. After integrating all pollutant-related targets, duplicate genes were removed to generate a nonredundant list of pollutant-associated target genes.

AD-associated genes were collected from five independent disease-gene association databases: GeneCards, OMIM, GWAS Catalog, MalaCards, and Open Targets. Genes identified in at least three of these five databases were defined as high-confidence AD-related genes. The resulting gene list was manually curated to remove duplicates and ensure biological relevance. The intersection between pollutant-associated genes and high-confidence AD-related genes was defined as the candidate pollutant–AD interaction target set for downstream analyses.

### 2.6 Functional enrichment analysis

Gene Ontology (GO) and Kyoto Encyclopedia of Genes and Genomes (KEGG) enrichment analyses were performed to explore the biological functions and signaling pathways of the shared pollutant–AD targets. GO enrichment included biological process, molecular function, and cellular component categories. KEGG pathway enrichment was used to identify signaling pathways potentially involved in pollutant-associated AD pathogenesis.

Enrichment analyses were performed using the R packages clusterProfiler, enrichplot, and org.Hs.eg.db. Adjusted *P* values were calculated using the Benjamini–Hochberg method. Terms with adjusted *P* values < 0.05 were considered statistically significant. Term–gene networks were generated to visualize relationships between enriched biological terms and corresponding genes.

### 2.7 Protein–protein interaction (PPI) network construction

The shared targets between air pollutant-associated genes and AD-related genes were imported into the STRING database for PPI network construction. A minimum required interaction score of 0.4, corresponding to medium confidence, was used as the threshold. The resulting PPI network was imported into Cytoscape software version 3.10.3 for visualization and topological analysis. All 19 shared genes were submitted to STRING.

**Figure 2:**
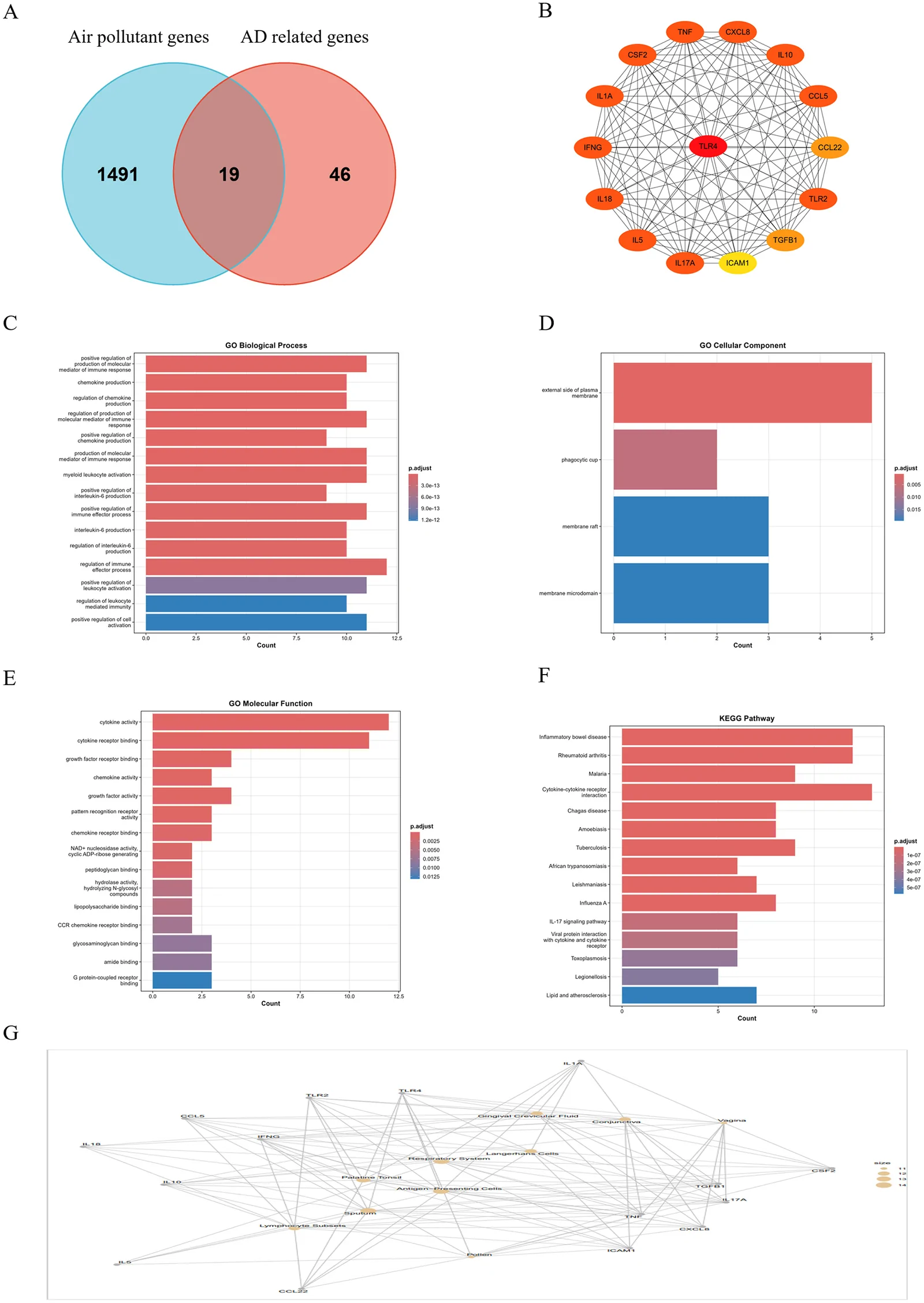
Integrated network toxicology analysis identifies immune-inflammatory targets linking air pollutants to atopic dermatitis. **(A)** Venn diagram showing the overlap between 1,510 pollutant-associated genes and 65 high-confidence AD-associated genes, yielding 19 shared targets. **(B)** PPI network showing the top 15 hub genes prioritized from the 19 shared targets by cytoHubba topological analysis. **(C)** GO biological process enrichment analysis of the shared targets. **(D)** GO cellular component enrichment analysis. **(E)** GO molecular function enrichment analysis. **(F)** KEGG pathway enrichment analysis of the shared targets. **(G)** Term–gene network showing the relationships between enriched pathways or biological terms and their associated genes.

Hub genes were identified using the cytoHubba plugin. Topological parameters, including node degree and Maximum Clique Centrality (MCC), were calculated to prioritize core targets within the network. The final PPI network and hub genes were used for downstream biological interpretation and integration with machine learning results. For visual clarity, Figure 2B displays the top 15 hub genes ranked by MCC and degree, whereas all 19 shared genes were retained as the input target set for downstream analyses.

### 2.8 Machine learning algorithms

To develop a robust diagnostic model linking pollutant-related genes to AD, we employed 15 machine learning algorithms: Lasso, Ridge, Elastic Net, Stepwise Generalized Linear Model, glmBoost, plsRglm, Random Forest, Generalized Boosted Regression Modeling, XGBoost, Support Vector Machine, Linear Discriminant Analysis, Quadratic Discriminant Analysis, K-nearest neighbors, Naive Bayes, and AdaBoost. Based on systematic combinatorial modeling strategies, a total of 175 predictive model combinations were explored for feature selection and classifier construction.

The training cohort was used for model development, whereas three independent GEO datasets were used for external validation. A stratified 10-fold cross-validation strategy was applied to maintain class balance across folds. Hyperparameter tuning was performed using grid search within the training data. Model performance was evaluated using the area under the receiver operating characteristic curve (AUC), sensitivity, and specificity. To compare overall performance across models, a composite score was calculated as follows: Total score = 0.5 × mean AUC + 0.25 × mean sensitivity + 0.25 × mean specificity. The model with the highest composite score across the training and validation cohorts was selected as the optimal classifier. The final diagnostic signature was then used for downstream nomogram construction, calibration analysis, decision curve analysis, and single-gene diagnostic evaluation. Stochastic model fitting and cross-validation were implemented using a random seed of 123.

### 2.9 *In silico* CCL22 perturbation analysis

To explore the potential functional role of CCL22 in AD skin, *in-silico* knockout analysis was performed using the scTenifoldKnk package (17). The AD skin single-cell expression matrix was used as input, and CCL22 was selected as the perturbation target. The algorithm constructed gene regulatory networks before and after virtual CCL22 knockout and identified genes predicted to be perturbed by CCL22 depletion. Differentially perturbed genes were ranked according to fold change, Z score, and adjusted *P* value. The top-ranked perturbed genes were visualized using volcano plots and bar plots. KEGG and Reactome enrichment analyses were then performed to identify biological pathways affected by CCL22 perturbation.

### 2.10 Animal model

C57 mice were used to establish a DNFB-induced AD-like dermatitis model (18). Mice were randomly divided into vehicle control and DNFB-treated groups. For sensitization, 100 μL of 0.1% DNFB solution was applied topically to shaved dorsal skin on day 0. Mice in the control group received vehicle treatment. Subsequently, mice were challenged with 50 μL of 0.1% DNFB on days 5, 8, 11, and 14. Skin tissues were collected on day 17 for molecular and histological analyses.

All animal experiments were performed according to the principles specified in the ‘Guide for the Care and Use of Laboratory Animals in China' with approval from the Animal Ethics Committee of Shanghai Jiao Tong University School of Medicine (A2026160).

### 2.11 Immunohistochemical staining

Skin tissues were fixed in 4% paraformaldehyde, dehydrated through graded ethanol, cleared, and embedded in paraffin. Paraffin sections were deparaffinized, rehydrated, and subjected to antigen retrieval in citrate antigen retrieval buffer (pH 6.0) using microwave heating for 20 min. Endogenous peroxidase activity was blocked using endogenous peroxidase blocking solution for 10 min at room temperature. Nonspecific binding was blocked with normal goat serum for 1 h at room temperature.

Sections were incubated overnight at 4 °C with a rabbit anti-MDC/CCL22 primary antibody (1:400; Abcam ab124768). After washing with PBS, sections were incubated with the corresponding secondary antibody for 30 min, followed by HRP–streptavidin incubation for 30 min. Immunoreactivity was visualized using a 3, 3′-diaminobenzidine substrate kit, and nuclei were counterstained with hematoxylin. Images were captured using a Nikon light microscope. The relative DAB-positive area or staining intensity was quantified using ImageJ software.

### 2.12 Quantitative real-time PCR

Total RNA was extracted from mouse skin tissues collected from mice in different experimental groups using the Tissue RNA Purification Kit (EZBioscience, China) following the manufacturer's instructions. RNA was reverse-transcribed into cDNA with the 4 × EZscript Reverse Transcription Mix II for qPCR (EZBioscience, China). Quantitative real-time PCR (qRT-PCR) was then carried out on a real-time PCR instrument using 2 × SYBR Green qPCR Master Mix (EZBioscience, China). Relative Ccl22 mRNA expression was normalized to the housekeeping gene Gapdh and calculated using the 2^−Δ*ΔCt*^ method. Ccl22 primer sequences were as follows: forward, CACCCTCTGCCATCACGTTTA; reverse, GGCACAGATATCTCGGTTCTTG. (Gapdh primers were provided by the kit or obtained commercially).

### 2.13 Measurement of skin CCL22 protein levels

Skin tissues were collected and homogenized in cold lysis buffer or phosphate-buffered saline containing protease inhibitors. After centrifugation, supernatants were collected for detection of CCL22 protein levels. Skin CCL22 concentrations were measured using a mouse CCL22 ELISA kit according to the manufacturer's instructions. Absorbance was measured using a microplate reader, and CCL22 concentrations were calculated according to the standard curve. Results were expressed as ng/mL or normalized to total protein concentration when applicable.

### 2.14 Protein structure preparation

Three-dimensional structures of target proteins were obtained from the Protein Data Bank or predicted using AlphaFold when experimentally resolved structures were unavailable (19). Protein structures were preprocessed before docking. Only the primary chain was retained when appropriate, and water molecules, ions, and unrelated heteroatoms were removed. Hydrogen atoms were added, and the processed protein structures were converted into PDBQT format using SailVina's built-in preprocessing module.

### 2.15 Ligand preparation and docking procedure

Ligand structures of structurally defined air pollutants were obtained from PubChem. All ligand structures were energy-minimized and converted into PDBQT format using Open Babel version 2.4.1 (20). Molecular docking was performed using AutoDock Vina version 1.2.3 (21). The docking box for each receptor was centered on the geometric center of the protein structure, with a 10 Å buffer added in each dimension.

High-throughput docking was performed using an in-house wrapper script, SailVina. Docking parameters were set as follows: exhaustiveness = 100, num_modes = 10, and energy_range = 3. For each pollutant–target pair, the docking pose with the lowest predicted binding affinity was retained for downstream analysis. Ligand–receptor binding interactions were visualized using PyMOL.

### 2.16 Interaction profiling

Protein–ligand complexes were uploaded to the Protein–Ligand Interaction Profiler web server to identify and annotate noncovalent interactions. Interaction types included hydrophobic contacts, hydrogen bonds, π-π stacking interactions, water bridges, and salt bridges when detected. Residue-level interaction information, including distances, angles, and atomic interaction details, was extracted. Representative pollutant–target complexes were visualized using PyMOL with transparent protein cartoons, highlighted ligand sticks, and labeled key residues.

### 2.17 Statistical analysis

All statistical analyses were performed using R software version 4.5.1. Continuous variables were expressed as the mean ± standard error of the mean or mean ± standard deviation, depending on data distribution. Comparisons between two groups were performed using Student's *t* test for normally distributed data or the Wilcoxon rank-sum test for nonnormally distributed data. For enrichment analyses, adjusted *P* values were calculated using the Benjamini–Hochberg method. ROC analysis was used to evaluate diagnostic performance, and AUC values were reported. For animal experiments, statistical significance was defined as a two-sided *P* value < 0.05. In addition to *P* values, effect measures were reported as Spearman correlation coefficients, AUC values, or fold-change and Z-score estimates, as appropriate to each analysis. Software versions are specified in the relevant methodological subsections.

## 3 Results

### 3.1 Epidemiological evidence linking air pollution and AD

A structured literature search identified epidemiological studies evaluating the impact of air pollution on AD risk or flare-ups (Supplementary Table S2). Across diverse populations and study designs, including time-series, case-crossover, and meta-analytic evidence, short-term increases in ambient air pollutants were generally associated with greater AD-related health care use and worsening disease activity. A recent meta-analysis including 42 studies from 14 countries showed that higher levels of PM_10_, SO_2_, and, with moderate certainty, PM_2.5_ and NO_2_, were associated with increased AD outpatient visits (22). Similarly, a large pediatric time-series study in Shanghai reported that short-term exposure to PM_2.5_, PM_10_, NO_2_, SO_2_, and CO was associated with increased outpatient visits for AD (23), whereas a case-crossover study in patients treated with dupilumab demonstrated a dose-dependent increase in the odds of AD flare with higher PM_10_, PM_2.5_, NO_X_, and NO_2_ exposure (24).

At the ecological level, pooled country-year correlation analyses in Supplementary Figure S1 (*n* = 6,324) revealed pollutant-specific associations between ambient air pollution and AD burden. Household air pollution (HAP) showed moderate-to-strong negative correlations with the age-standardized prevalence rate (ASPR; rho = −0.608, *P* < 0.001), age-standardized incidence rate (ASIR; rho = −0.605, *P* < 0.001), and age-standardized DALY rate (ASDR; rho = −0.623, *P* < 0.001). PM pollution exhibited similar inverse correlations with ASPR (rho = −0.514, *P* < 0.001), ASIR (rho = −0.551, *P* < 0.001), and ASDR (rho = −0.525, *P* < 0.001). In contrast, NO_2_ showed consistent positive correlations with ASPR (rho = 0.464, *P* < 0.001), ASIR (rho = 0.397, *P* < 0.001), and ASDR (rho = 0.477, *P* < 0.001), while ozone demonstrated weaker but statistically significant positive correlations with ASPR (rho = 0.073, *P* < 0.001), ASIR (rho = 0.026, *P* = 0.038), and ASDR (rho = 0.082, *P* < 0.001). Collectively, these findings support a meaningful but heterogeneous association between air pollution and AD, suggesting that the direction and magnitude of the relationship may vary across pollutant species and epidemiological scales.

### 3.2 Toxicological profiling of selected air pollutants

To assess the potential skin-related toxicity of environmental chemicals, seven representative air pollutants were selected for molecular descriptor evaluation, including benzene, CO, NO, NO_2_, O_3_, SO_2_, and toluene. Notably, CO, O_3_, and SO_2_ showed the highest skin sensitization scores (1.00, 1.00, and 0.99, respectively), while NO and O_3_ exhibited elevated activation of the oxidative stress response element (SR-ARE) pathway (0.52 and 0.43, respectively). Benzene and NO demonstrated moderate activity in the matrix metalloproteinase-related (SR-MMP) pathway, which has been implicated in tissue remodeling and inflammation (Supplementary Table S3). These results provide computational support for the immunotoxic and skin-damaging potential of the selected pollutants.

### 3.3 Identification of shared targets between air pollutants and atopic dermatitis

To characterize the molecular overlap between pollutant-associated and AD-related gene sets, we intersected 1,510 nonredundant pollutant-associated genes with 65 high-confidence AD-associated genes. Complete gene lists are provided in Supplementary Tables S4 and S5, respectively. The intersection yielded 19 shared genes: CCL22, CCL5, CSF2, CXCL8, F2RL1, HRH4, ICAM1, IFNG, IL10, IL17A, IL18, IL1A, IL4R, IL5, NOD2, TGFB1, TLR2, TLR4, and TNF (Figure 2A; Supplementary Table S6). These genes defined a pollutant-prioritized immune-inflammatory candidate set for downstream analyses.

All 19 shared genes were submitted to STRING, and Figure 2B displays the top 15 hub genes ranked by Maximum Clique Centrality and degree using cytoHubba. The densely connected hub subnetwork was dominated by cytokines, chemokines, and innate immune regulators, indicating coordinated immune-inflammatory signaling among the shared targets.

GO biological process analysis showed enrichment in chemokine production, regulation of molecular mediator production, myeloid leukocyte activation, interleukin-6 production, and immune effector regulation (Figure 2C). Cellular component terms included the external side of the plasma membrane, phagocytic cup, membrane raft, and membrane microdomain (Figure 2D). Molecular function terms included cytokine and chemokine activity, receptor binding, growth factor activity, and pattern-recognition receptor activity (Figure 2E).

KEGG analysis highlighted cytokine–cytokine receptor interaction, IL-17 signaling, inflammatory bowel disease, rheumatoid arthritis, and infection-related pathways sharing cytokine and innate immune genes (Figure 2F). These disease-labeled terms largely reflected overlapping TNF-, interleukin-, chemokine-, and Toll-like receptor-associated networks. The term–gene network showed that TNF, IL10, IL17A, IL18, IFNG, CCL5, CCL22, CXCL8, TLR2, TLR4, ICAM1, and TGFB1 contributed to multiple enriched terms (Figure 2G). Overall, the shared target set was characterized by cytokine signaling, TLR-related innate immune activation, and chemokine-dependent immune-cell recruitment.

### 3.4 Machine learning identified a robust air pollutant–associated gene signature for atopic dermatitis

**Figure 3:**
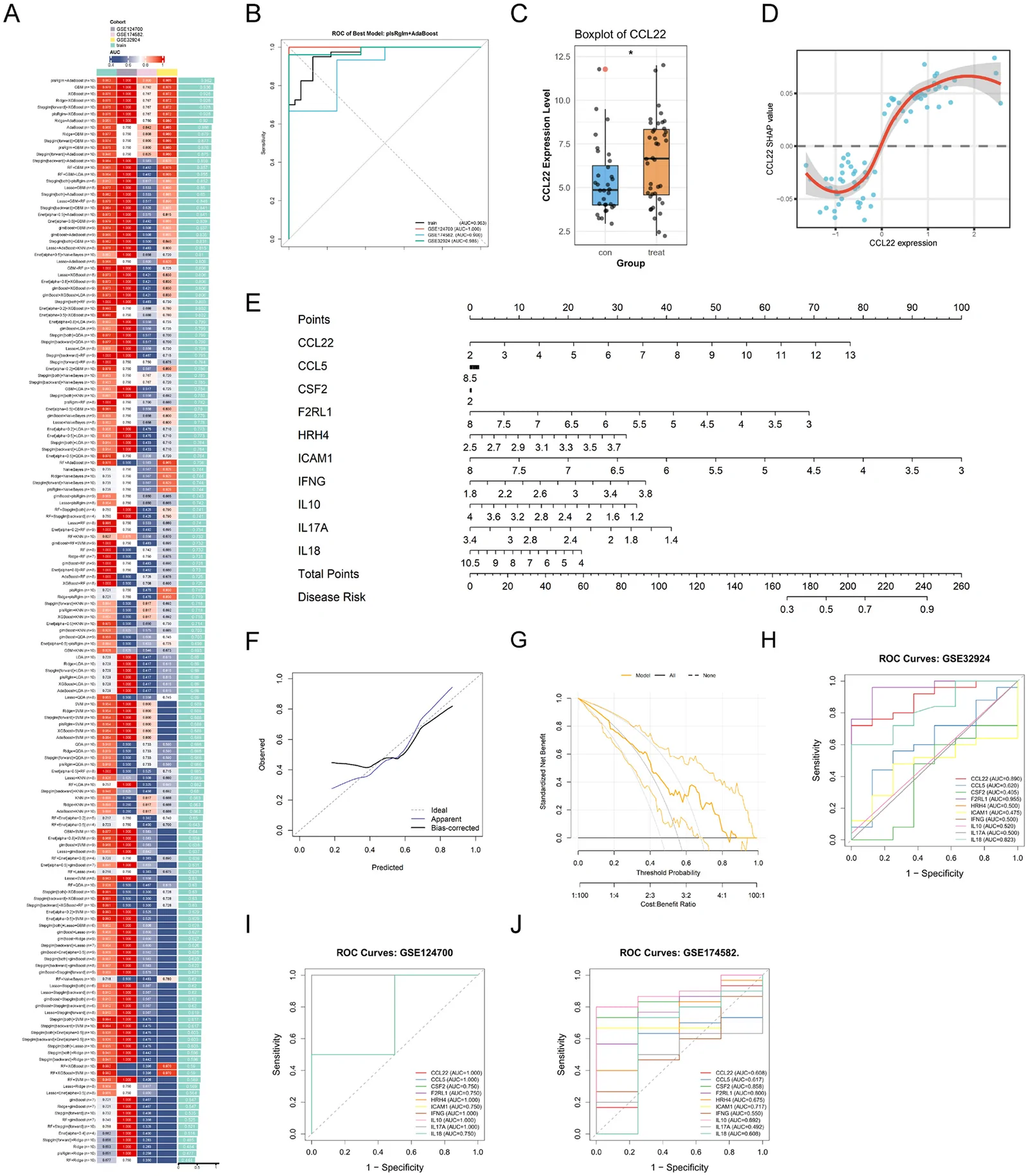
Machine learning identifies a pollutant-related diagnostic gene signature for atopic dermatitis. **(A)** Performance heatmap of multiple machine learning models across the training cohort and three independent validation datasets. **(B)** ROC curves of the optimal model in the training and validation cohorts. **(C)** Boxplot showing differential expression of CCL22 between control and AD samples. **(D)** SHAP dependence plot showing the contribution of CCL22 expression to AD prediction. **(E)** Nomogram constructed using the selected pollutant-related diagnostic genes for individualized AD risk prediction. **(F)** Calibration curve evaluating the agreement between predicted and observed disease probabilities. **(G)** Decision curve analysis assessing the clinical net benefit of the diagnostic model across different threshold probabilities. **(H–J)** ROC curves evaluating the diagnostic performance of individual model genes in independent validation datasets.

To prioritize robust diagnostic biomarkers linking pollutant-associated genes to AD, we implemented a comprehensive machine learning pipeline encompassing 15 algorithms systematically combined to generate 175 predictive models. The performance heatmap showed heterogeneous predictive performance across the training cohort and three independent validation datasets (Figure 3A; Supplementary Table S7). Among these models, the plsRglm + AdaBoost model showed the most consistent overall performance and achieved the highest composite score based on the mean AUC, sensitivity, and specificity across datasets (Supplementary Table S8). Because 175 model combinations were screened, the selected classifier was interpreted as exploratory rather than definitive.

ROC analysis further confirmed the strong discriminative ability of the optimal model. The plsRglm + AdaBoost model achieved an AUC of 0.963 in the training cohort and maintained favorable external validation performance, with AUC values of 1.000 in GSE124700, 0.909 in GSE174582, and 0.966 in GSE32924 (Figure 3B). These findings indicate that the pollutant-related gene signature has stable diagnostic potential for distinguishing AD samples from controls across independent transcriptomic cohorts; however, the AUC of 1.000 in GSE124700 should be interpreted cautiously because this external cohort contained only four samples (two AD and two controls), and AUC confidence intervals were not estimated.

The final diagnostic signature consisted of 10 genes: CCL22, CCL5, CSF2, F2RL1, HRH4, ICAM1, IFNG, IL10, IL17A, and IL18. The expression patterns and model contributions of the signature genes were further evaluated. Except for CCL22, most genes in the diagnostic panel did not show significant univariate differences between control and AD samples (Supplementary Figure S2A). However, SHAP analysis demonstrated that these genes still contributed to model prediction in a heterogeneous manner (Supplementary Figure S2B). Mean absolute SHAP ranking identified ICAM1, F2RL1, IL10, IL18, CSF2, and CCL22 as the main contributors to the optimal model (Supplementary Figure S2C), suggesting that the pollutant-related diagnostic signature captured combinatorial immune-inflammatory information rather than relying solely on individually differentially expressed genes.

Among the signature genes, CCL22 showed significantly higher expression in AD samples than in control samples (Figure 3C), suggesting that CCL22 may represent a candidate chemokine-related marker associated with AD immune activation. SHAP dependence analysis further supported the predictive contribution of CCL22. As CCL22 expression increased, its SHAP value shifted from negative to positive, indicating that higher CCL22 expression contributed positively to the predicted probability of AD classification (Figure 3D). This nonlinear pattern suggests that CCL22 may exert a threshold-like contribution to model prediction rather than a simple linear effect.

Based on the ten-gene signature, we constructed a nomogram to estimate individualized AD risk (Figure 3E). The nomogram integrated chemokine-related genes, cytokine-associated genes, inflammatory mediators, and immune-response genes, providing a visual tool for AD risk stratification. Calibration analysis showed that the predicted probabilities were generally consistent with the observed outcomes, and the bias-corrected curve followed the ideal reference line with acceptable agreement (Figure 3F). Decision curve analysis further demonstrated that the diagnostic model provided a higher standardized net benefit than the “all” or “none” strategies across a broad range of threshold probabilities (Figure 3G), supporting its potential clinical utility.

We next evaluated the diagnostic performance of individual genes in the independent validation cohorts. In GSE32924, several genes showed moderate-to-strong discriminative ability, particularly F2RL1, CCL22, and IL18 (Figure 3H). In GSE124700, multiple genes, including CCL22, CCL5, HRH4, IFNG, IL10, and IL17A, showed excellent AUC values (Figure 3I). In GSE174582, genes such as CSF2, F2RL1, IL10, and IL18 showed relatively strong predictive performance (Figure 3J). Collectively, these results suggest that the CCL22 was prioritized for downstream analyses because it combined significant differential expression, contribution to the multigene model, diagnostic performance in external cohorts, and established biological relevance to CCR4-associated AD inflammation; it was not selected solely on network centrality or a single ranking metric.

### 3.5 Bulk immune-signature analysis associates CCL22 with AD-related immune activation

**Figure 4:**
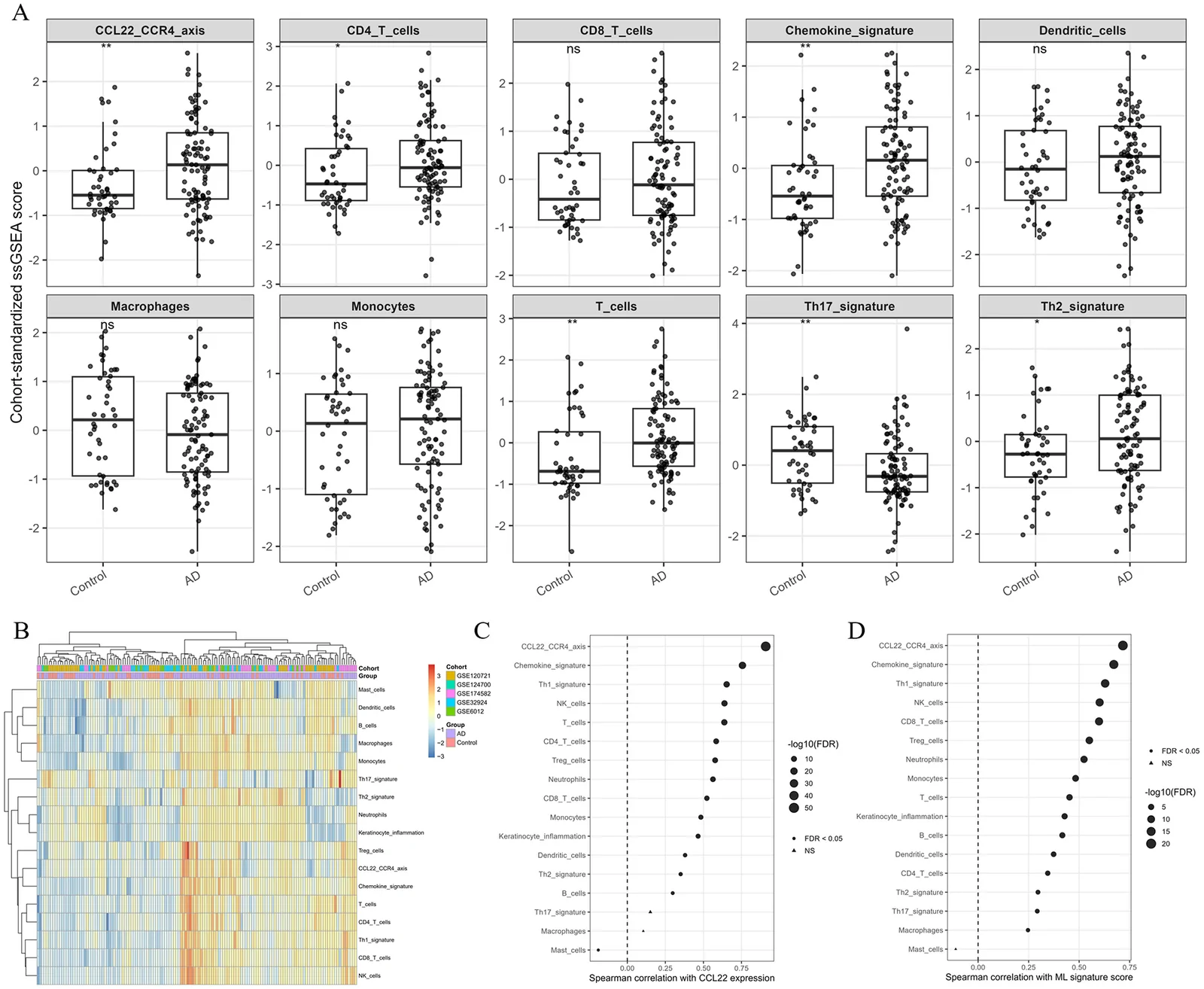
Bulk immune-signature analysis associates CCL22 and the pollutant-prioritized AD signature with a chemokine-related immune microenvironment. **(A)** Boxplots comparing cohort-standardized ssGSEA scores of selected immune and inflammatory signatures between control and AD skin samples. Significant differences and non-significant comparisons are indicated in each panel. **(B)** Heatmap of cohort-standardized ssGSEA scores across bulk transcriptomic samples, annotated by disease group and GEO cohort. **(C)** Bubble plot showing Spearman correlations between CCL22 expression and immune infiltration or inflammatory signatures. Bubble size represents –log10(FDR), and point shape indicates statistical significance.**(D)** Bubble plot showing Spearman correlations between the machine-learning-derived pollutant-related AD signature score and immune infiltration or inflammatory signatures.

Because CCL22 emerged as a key gene in the pollutant-related diagnostic model, we next examined whether CCL22 was associated with AD-related immune infiltration and chemokine activation in bulk transcriptomic datasets. ssGSEA-based immune scoring showed that the CCL22–CCR4 axis was significantly increased in AD samples compared with controls (Figure 4A). AD samples also showed elevated chemokine signature, T-cell score, and Th2 signature, consistent with enhanced chemokine-mediated immune recruitment and adaptive immune activation in AD lesions. In contrast, several immune cell scores, including CD4 T cells, CD8 T cells, dendritic cells, macrophages, and monocytes, showed variable but non-significant differences between AD and control samples, suggesting that pathway-level immune activation may be more robust than individual cell-type estimates in bulk transcriptomic data.

Unsupervised heatmap visualization of immune infiltration and inflammatory signatures further showed distinct immune microenvironment patterns across samples (Figure 4B). AD samples tended to cluster with higher scores for chemokine-related and T-cell-associated signatures, including the CCL22–CCR4 axis, chemokine signature, T cells, Th2 signature, and keratinocyte inflammation. These findings indicate that AD skin is characterized by a coordinated immune activation program involving chemokine signaling, T-cell responses, and epithelial inflammation.

Correlation analysis further demonstrated that CCL22 expression was strongly and positively associated with the CCL22–CCR4 axis, supporting the internal consistency of this chemokine pathway (Figure 4C). CCL22 expression was also positively correlated with the chemokine signature, Th1 signature, NK cell score, T-cell score, CD4 T-cell score, Treg score, neutrophil score, CD8 T-cell score, monocyte score, keratinocyte inflammation, and dendritic cell score. These results suggest that CCL22 expression reflects a broad immune-activated state in AD rather than an isolated chemokine signal.

Similarly, the machine learning signature score showed positive correlations with multiple immune and inflammatory features, including the CCL22–CCR4 axis, chemokine signature, Th1 signature, NK cells, CD8 T cells, Tregs, neutrophils, monocytes, T cells, keratinocyte inflammation, B cells, dendritic cells, CD4 T cells, Th2 signature, and Th17 signature (Figure 4D). Together, these results indicate that both CCL22 expression and the pollutant-related diagnostic signature are closely linked to AD-associated immune activation. These findings provide bulk transcriptomic evidence that CCL22 expression is associated with a broader chemokine-centered immune state in AD skin.

### 3.6 Single-cell transcriptomic analysis localizes CCL22 expression mainly to dendritic cell and macrophage populations

**Figure 5:**
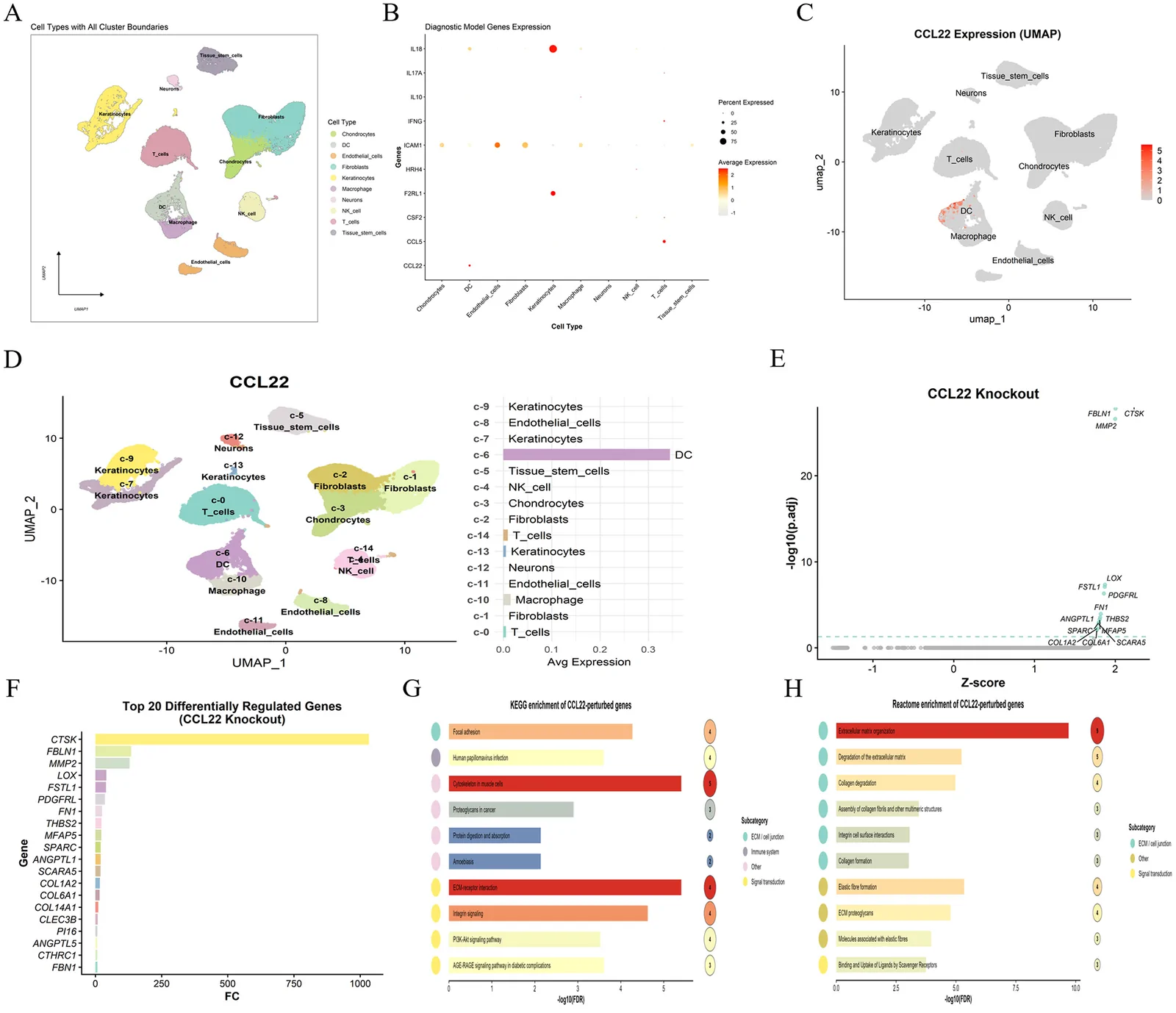
Single-cell transcriptomic analysis identifies dendritic cells and macrophages as major cellular sources of CCL22 in atopic dermatitis skin. **(A)** UMAP visualization of single-cell transcriptomic profiles from AD skin, showing major skin-resident and immune cell populations, including keratinocytes, fibroblasts, chondrocytes, endothelial cells, tissue stem cells, neurons, T cells, NK cells, macrophages, and dendritic cells. **(B)** Dot plot showing the expression distribution of pollutant-related diagnostic model genes across annotated cell types. Dot size represents the percentage of cells expressing each gene, and color intensity represents the average expression level. **(C)** Feature plot showing the distribution of CCL22 expression within the UMAP embedding. CCL22 expression was mainly concentrated in dendritic cell/macrophage-enriched regions. **(D)** Cluster-level UMAP annotation and quantitative comparison of average CCL22 expression across clusters/cell types. CCL22 showed the highest average expression in dendritic cells, with lower expression in macrophages and selected immune cell populations. **(E)** Volcano plot showing genes predicted to be perturbed after *in silico* CCL22 network perturbation. **(F)** Bar plot showing the top genes predicted to be perturbed after virtual CCL22 perturbation, including extracellular matrix- and stromal remodeling-related genes. **(G)** KEGG enrichment analysis of CCL22-perturbed genes, highlighting pathways related to cytokine signaling, IL-17 signaling, ECM–receptor interaction, focal adhesion, and inflammation-associated processes. **(H)** Reactome enrichment analysis of CCL22-perturbed genes, showing enrichment in extracellular matrix organization, degradation of the extracellular matrix, collagen degradation, collagen fibril assembly, collagen formation, elastic fiber formation, and ECM proteoglycans.

To further define the cellular source and functional relevance of CCL22 in AD skin, we analyzed single-cell transcriptomic profiles from AD skin tissues. Uniform manifold approximation and projection (UMAP) visualization identified major skin-resident and immune cell populations, including keratinocytes, fibroblasts, chondrocytes, endothelial cells, tissue stem cells, neurons, T cells, NK cells, macrophages, and dendritic cells (Figure 5A). Dot plot analysis of the pollutant-related diagnostic genes revealed distinct cell-type-specific expression patterns. Among these genes, CCL22 showed a restricted expression profile and was mainly detected in immune cell populations, particularly dendritic cells and macrophages (Figure 5B).

Feature plot visualization further confirmed that CCL22 expression was concentrated in the dendritic cell/macrophage-enriched region of the UMAP space, rather than being broadly distributed across keratinocytes, fibroblasts, endothelial cells, or other stromal populations (Figure 5C). Cluster-level annotation and quantitative expression comparison further supported this pattern. CCL22 expression was highest in the dendritic cell cluster, with lower but detectable expression in macrophages and selected immune cell populations, whereas most epithelial and stromal cell populations showed minimal expression (Figure 5D). These findings suggest that CCL22 is mainly derived from antigen-presenting and myeloid immune cell populations in AD skin.

To generate network-level hypotheses about CCL22, we performed *in silico* virtual perturbation using the single-cell expression matrix. Virtual CCL22 perturbation predicted marked network-level transcriptomic changes, as shown by the volcano plot of differentially perturbed genes (Figure 5E). The most strongly predicted perturbed genes included several extracellular matrix- and stromal remodeling-related genes, such as CTSK, FBLN1, MMP2, LOX, PDGFRL, FSTL1, FBN1, THBS2, MFAP5, SPARC, ANGPTL1, SCARA5, COL1A1/COL6A1-related genes, CLEC3B, PI16, and CTHRC1 (Figure 5F). These genes are closely associated with collagen organization, matrix degradation, fibroblast activation, and tissue structural remodeling, supporting a hypothesis-generating association between CCL22-related regulatory networks and stromal remodeling programs in AD lesions.

Functional enrichment analysis of the CCL22-perturbed genes further supported this interpretation. KEGG analysis showed enrichment in pathways related to cytokine–cytokine receptor interaction, IL-17 signaling pathway, ECM–receptor interaction, focal adhesion, and inflammation-associated pathways (Figure 5G). Reactome enrichment analysis revealed prominent enrichment in extracellular matrix organization, degradation of the extracellular matrix, collagen degradation, assembly of collagen fibrils and other multimeric structures, collagen formation, elastic fiber formation, and ECM proteoglycans (Figure 5H). Together, the single-cell analysis localizes CCL22 expression mainly to dendritic cells and macrophages, whereas the virtual perturbation identifies predicted associations with chemokine and extracellular-matrix programs.

### 3.7 DNFB-induced AD-like dermatitis confirms disease-associated CCL22 upregulation in mouse skin

**Figure 6:**
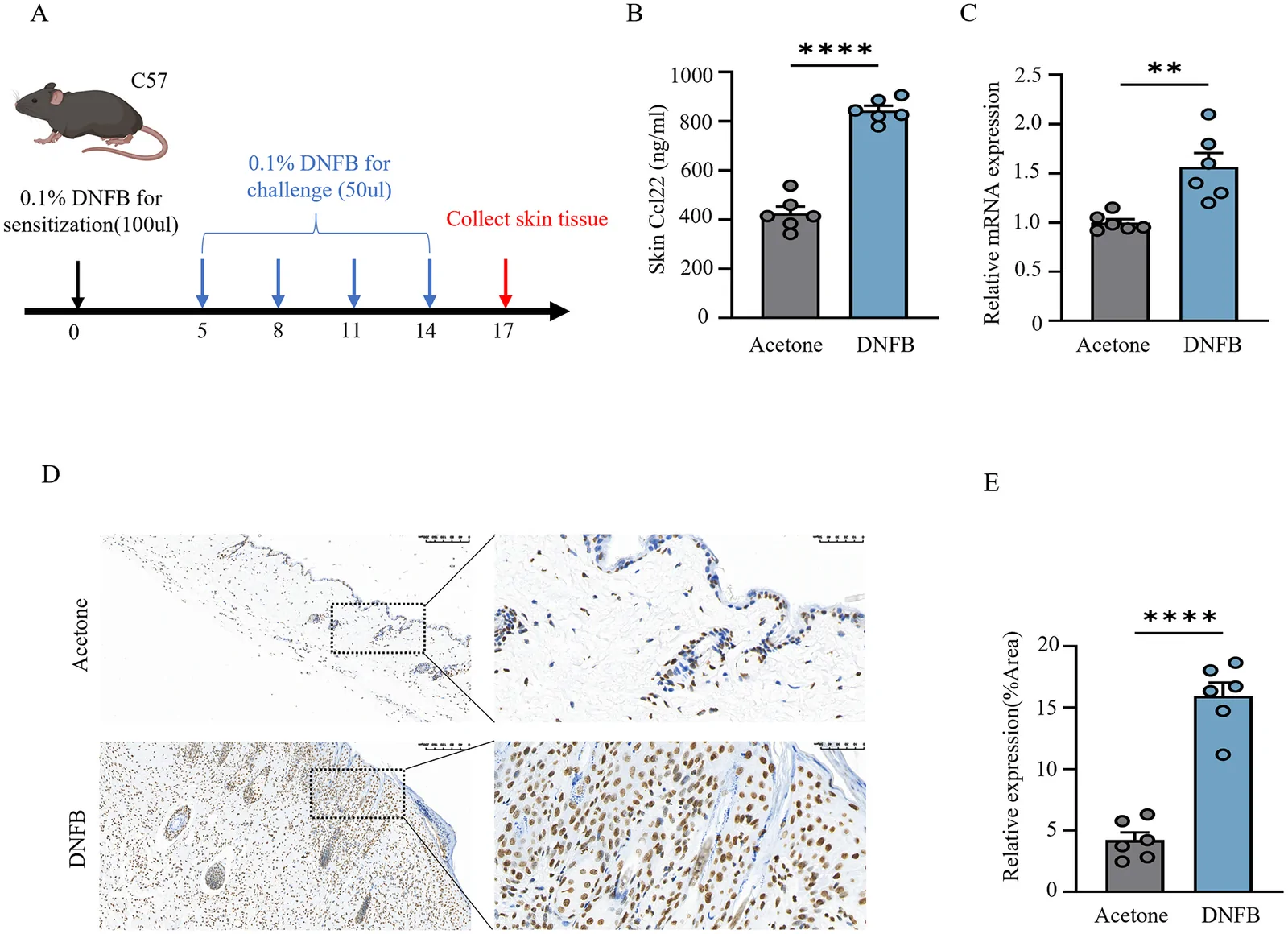
DNFB-induced atopic dermatitis-like skin inflammation upregulates CCL22 expression in mouse skin. **(A)** Schematic diagram of the DNFB-induced AD-like dermatitis model. C57 mice were sensitized and repeatedly challenged with 0.1% DNFB, and skin tissues were collected for molecular and histological analyses **(B)** Quantification of skin CCL22 protein levels in acetone-treated control and DNFB-treated mice. **(C)** Quantitative PCR analysis of Ccl22 mRNA expression in control and DNFB-induced lesional skin. **(D)** Representative immunohistochemical staining images showing CCL22 expression in mouse skin sections from control and DNFB-treated groups. Enlarged regions highlight CCL22-positive staining in lesional skin. **(E)** Quantification of CCL22-positive staining area in skin tissues Data are shown as mean ± SEM with individual biological replicates; *n* = 6 mice per group. **P < 0.01, ***P < 0.001, and ****P < 0.0001.

To assess whether CCL22 expression is increased in AD-like inflammatory skin, we established a DNFB-induced dermatitis model in C57 mice. Mice were repeatedly challenged with 0.1% DNFB on days 5, 8, 11, and 14, and skin tissues were collected on day 17 for molecular and histological analyses (Figure 6A).

Compared with the acetone-treated control group, DNFB-challenged mice showed a marked increase in skin CCL22 protein levels (Figure 6B). Consistently, Ccl22 mRNA expression was also significantly upregulated in DNFB-induced lesional skin (Figure 6C), indicating that CCL22 was transcriptionally activated during AD-like cutaneous inflammation.

Immunohistochemical staining further confirmed the increased CCL22 expression in DNFB-treated skin. In the acetone group, CCL22-positive staining was weak and sparsely distributed. In contrast, DNFB-induced lesions displayed stronger CCL22 immunoreactivity, mainly localized in the inflammatory cell-rich dermal compartment and around the thickened epidermal region (Figure 6D). Quantitative analysis showed that the relative CCL22-positive area was significantly higher in DNFB-treated skin than in control skin (Figure 6E).

Together, these results demonstrate that CCL22 is significantly upregulated at both the mRNA and protein levels in DNFB-induced AD-like skin lesions. This experiment supports the biological relevance of CCL22 in AD-like cutaneous inflammation.

### 3.8 Exploratory molecular docking predicts potential pollutant–target poses

**Figure 7:**
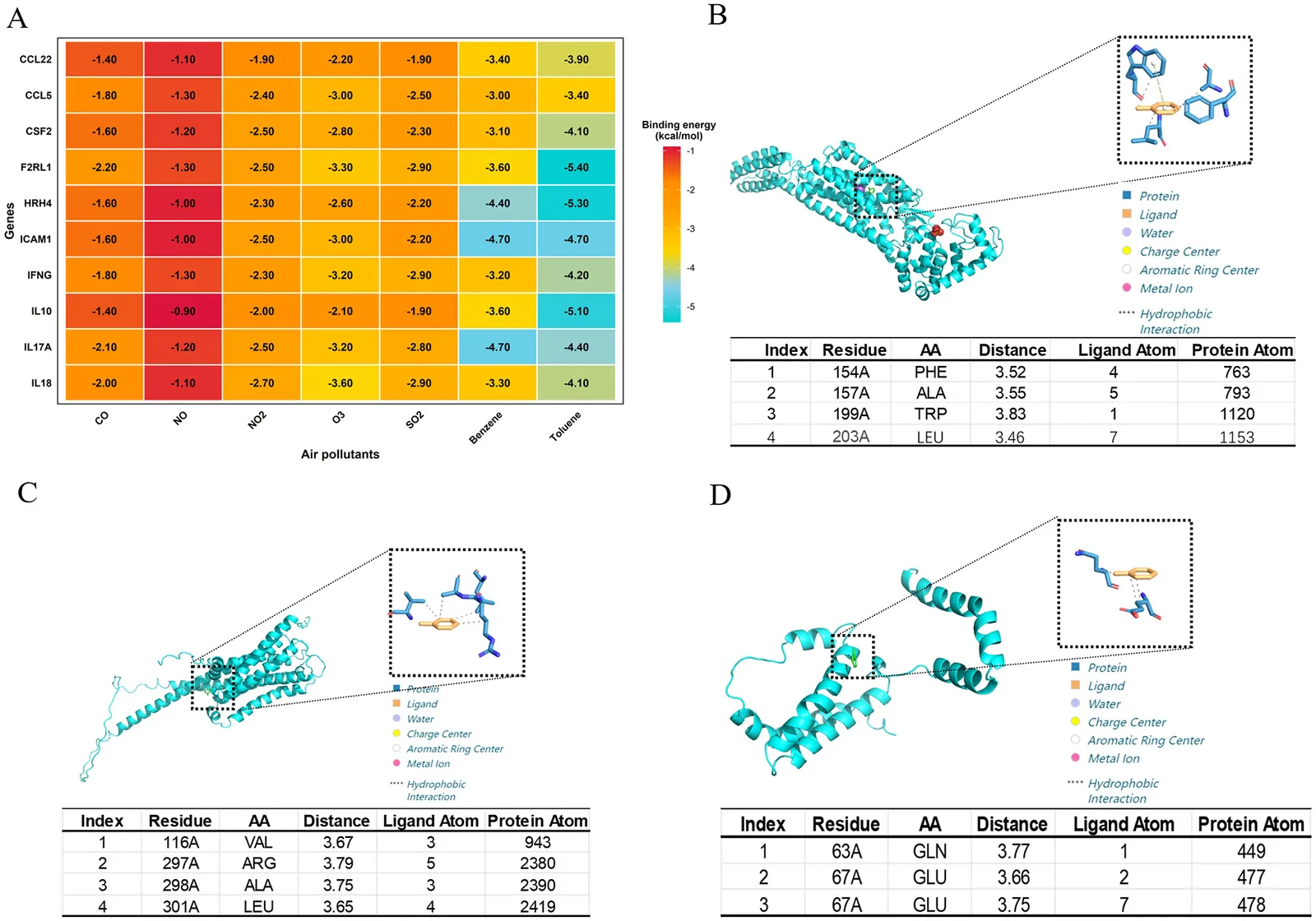
Exploratory molecular docking predicts potential low-energy poses between toluene and AD-related immune targets. **(A)** Heatmap of docking scores (kcal/mol) between seven structurally defined pollutants and 10 AD-related immune targets. **(B–D)** Representative predicted docking poses of toluene with F2RL1, HRH4, and IL10, respectively. More negative scores indicate more favorable predicted poses and do not represent experimentally measured binding affinities.

Exploratory molecular docking was performed between seven structurally defined pollutants and 10 AD-related targets to generate structural hypotheses for future experimental investigation. The docking score matrix is presented in Figure 7A, where lower scores indicate more favorable predicted poses rather than experimentally measured binding affinities. Toluene showed docking scores below −5.0 kcal/mol for several targets, including HRH4, IL10, and IL17A.

Three representative predicted docking poses were visualized in detail. Toluene showed docking scores of −5.4, −5.3, and −5.1 kcal/mol with F2RL1, HRH4, and IL10, respectively (Figures 7B–D). These computational results identify pollutant–target pairs that may warrant further investigation; however, they do not demonstrate direct physical binding, target modulation, or functional effects and require validation using biochemical and cellular assays.

## 4 Discussion

In this study, we integrated epidemiological evidence, network toxicology, transcriptomic machine learning, single-cell analysis, *in silico* perturbation, experimental validation, and molecular docking to characterize candidate molecular associations between air pollutant-related targets and AD. This integrated framework was designed to move beyond conventional single-pollutant or single-target toxicology and to prioritize shared molecular signatures associated with environmental exposure and immune dysregulation in AD. A similar multi-layered systems toxicology strategy has recently been applied to psoriasis, supporting the feasibility of combining pollutant-associated target mining, disease-related gene integration, pathway enrichment, machine learning, and molecular docking to investigate environmental immunotoxicity in inflammatory skin diseases (25). In the present AD-focused study, CCL22 emerged as a recurrent chemokine-related candidate associated with lesional immune–stromal remodeling.

Air pollution has increasingly been recognized as an environmental contributor to AD development and exacerbation. Epidemiological and mechanistic studies have linked exposure to particulate matter, nitrogen oxides, sulfur dioxide, ozone, carbon monoxide, traffic-related pollutants, and volatile organic compounds with AD risk, symptom aggravation, and health care utilization (4, 26). Recent systematic review and meta-analytic evidence further supports the association between air pollutants and AD burden, particularly for short-term exacerbation and outpatient visits (22). Mechanistically, air pollutants may contribute to AD through oxidative stress, epidermal barrier disruption, aryl hydrocarbon receptor (AhR)-dependent signaling, neuroimmune activation, and inflammatory pathway induction (8, 27). PM2.5 has been shown to induce TNF-α-dependent filaggrin deficiency through AhR signaling, resulting in impaired skin barrier function (8). In parallel, AhR activation can link air pollution to AD-like inflammation and itch through induction of the neurotrophic factor artemin (27). These findings provide biological plausibility for pollutant-associated AD exacerbation, but they do not establish a pollutant-specific pathway in the transcriptomic cohorts analyzed here.

The detrimental effects of environmental pollutants also extend to other allergic diseases involving barrier tissues. In an experimental asthma model, early-life and perinatal exposure to hypochlorous acid aggravated airway inflammation, accompanied by reduced gut microbial diversity and enhanced arachidonic acid metabolism; taurine supplementation attenuated these effects (28). In allergic rhinitis, a multi-omic study of 505 participants linked early-life exposure to several air toxics with disease development and identified nasal DNA methylation and gene-expression pathways involving respiratory ciliary function, mast-cell activation, pro-inflammatory TGF-β1 signaling, and regulation of myeloid immune cells (29). Together, these studies suggest that pollutant-related allergic diseases may share epithelial-barrier, innate immune, and type 2 inflammatory processes across the skin and respiratory mucosa, although the relevant exposures and tissue-specific causal pathways may differ among AD, asthma, and allergic rhinitis.

The enrichment of the shared target set in cytokine-, chemokine-, Toll-like receptor-, IL-17-, and extracellular-matrix-related pathways indicates convergence of database-derived pollutant targets and AD-associated genes on immune and stromal programs. This is consistent with the current understanding of AD as a heterogeneous epithelial–immune disease involving type 2 inflammation, Th1-, Th17-, Th22-, innate immune-, and chemokine-mediated responses rather than a purely Th2-restricted disorder (1, 3). Therefore, the identification of CCL22 in the present analysis is biologically plausible. CCL22, also known as macrophage-derived chemokine, is a ligand for CCR4 and has been implicated in the recruitment and spatial organization of CCR4-positive T cells within inflamed skin (30). In human inflamed skin, the MDC/CCL22–CCR4 system participates in the formation of T lymphocyte–dendritic cell clusters, indicating its role in local immune-cell organization and antigen-presenting cell–T-cell interactions.

CCL22 has also been repeatedly associated with AD disease activity. Serum MDC/CCL22 levels are closely related to AD disease activity, and both Th2- and Th1-associated chemokines, including TARC/CCL17, MDC/CCL22, and Mig/CXCL9, are elevated in sera from patients with AD (31, 32). In addition, CCL22 produced by monocyte-derived dendritic cells reflects disease activity in AD, supporting dendritic cells as an important cellular source of CCL22 in the AD inflammatory milieu (33). In the present study, single-cell transcriptomic analysis localized CCL22 predominantly to dendritic cells and macrophages in AD skin. However, CCL22 is an established AD-associated chemokine, and its elevation should not be interpreted as specific evidence of pollution-associated disease. Because the analyzed GEO cohorts lacked individual-level or regionally harmonized pollution exposure data, the present study cannot determine whether CCL22 expression differs between AD occurring in high- vs. low-pollution settings. CCL22 should therefore be interpreted here as an established AD chemokine re-prioritized within a pollutant-informed analytical framework. Thus, the present data do not distinguish pollution-associated AD from AD occurring in low-pollution settings.

Future studies should extend these observations using spatially resolved and clinically annotated datasets. Spatial transcriptomics combined with CellChat, CellPhoneDB, or related ligand–receptor inference frameworks could test whether dendritic cell- or macrophage-derived CCL22 is spatially associated with CCR4-positive T-cell populations and could directly compare the relative contributions of the CCL17–CCR4 and CCL22–CCR4 axes. These tools infer cell-to-cell communication from transcriptomic data and do not model chemical pollutant–host protein interactions; controlled exposure systems would therefore still be required. Protein-level validation in human lesional skin and serum, together with correlations with clinical severity and measured pollutant exposure, would further establish translational relevance. It will also be important to determine whether CCL22 changes during treatment with type 2-targeted biologics, including dupilumab, which blocks IL-4Rα-mediated IL-4/IL-13 signaling, and the IL-13-targeting antibodies tralokinumab and lebrikizumab (34–36). The present study does not establish CCL22 as a treatment-response biomarker.

Virtual CCL22 perturbation predicted network-level changes involving extracellular-matrix and stromal-remodeling genes, including DCN, C1R, FBLN1, ITGBL1, COL6A1, VCAN, MMP2, FBN1, CD248, SCARA5, LOX, DPT, ANGPTL1, CTSK, PDGFRL, MFAP5, SEMA3C, and THBS2. The scTenifoldKnk framework provides an efficient virtual knockout strategy to infer gene function through single-cell gene regulatory network perturbation (17). Therefore, these results should be interpreted as predictive network-level evidence rather than direct genetic knockout evidence. The enrichment of ECM-related genes after virtual perturbation therefore identifies a testable hypothesis of association between CCL22-related regulatory networks and immune–stromal crosstalk. This observation is particularly relevant because chronic AD is characterized by recurrent inflammation, epidermal thickening, dermal immune infiltration, and tissue remodeling.

The DNFB-induced AD-like dermatitis mouse model provided orthogonal validation of disease-associated CCL22 upregulation in lesional skin. Validation at the mRNA and protein levels supports the relevance of CCL22 to AD-like inflammation but not to pollution-specific regulation. However, DNFB-induced dermatitis does not fully recapitulate real-world pollutant exposure, and the present mouse experiment validates CCL22 activation in AD-like inflammation rather than directly proving pollutant-induced CCL22 regulation. Future studies should therefore incorporate controlled exposure models using PM2.5, NO_2_, ozone, benzene, toluene, or pollutant mixtures in keratinocytes, dendritic cells, macrophages, 3D skin equivalents, and AD-like mouse models. In addition, CCL22 neutralization or CCR4-axis blockade would be valuable to determine whether the CCL22 pathway causally mediates pollutant-induced AD exacerbation.

Exploratory molecular docking generated structural hypotheses for selected pollutant–target pairs but did not provide experimental evidence of binding or target modulation. PubChem was used to obtain chemical structures and identifiers, ADMETlab 3.0 was used to predict ADMET and toxicity-related descriptors, Open Babel was used for ligand structure conversion, and AutoDock Vina was used for docking analysis (20, 21, 37, 38). These computational tools are widely used for chemical informatics, toxicity prediction, and ligand–receptor docking. However, docking analysis should be interpreted as hypothesis-generating rather than causal evidence. The most biologically relevant pollutant–target interactions should be further validated using cellular exposure assays, ligand–receptor binding assays, and pathway-specific perturbation experiments.

Several limitations should be acknowledged. First, pollutant exposure was operationalized through CTD-derived chemical–gene annotations rather than measured concentrations, exposure duration, internal dose, or exposure–response relationships, and no exposure-resolved human transcriptomic cohort was analyzed. These curated targets may also be biased toward well-studied chemicals and genes. Second, particulate matter and VOC mixtures cannot be represented by a single molecular structure, while small reactive gases are poorly suited to standard docking assumptions. Third, the bulk datasets differed in platform, lesion status, disease severity, treatment history, and sample size. Screening 175 model combinations may introduce model-selection optimism; formal feature-stability analysis, bootstrap confidence intervals, and permutation testing were not performed, and the AUC of 1.000 in GSE124700 is particularly uncertain because that cohort contained only four samples. Fourth, bulk ssGSEA scores reflect relative transcriptomic enrichment and cannot distinguish cellular abundance from activation or tissue-composition effects. Fifth, scTenifoldKnk predicts regulatory consequences and is not equivalent to CRISPR-mediated knockout or functional inhibition. Finally, the DNFB model validates disease-associated CCL22 upregulation but not pollutant-induced regulation; controlled exposure and CCL22/CCR4 functional intervention will be required to test causality. In addition, spatial transcriptomics, formal ligand–receptor communication analysis, and protein-level validation in human skin or serum were not performed.

In conclusion, this integrated analysis prioritizes CCL22 as a candidate pollutant-informed immune biomarker associated with AD. Its selection was supported by convergent evidence from the multigene model, differential expression, immune-signature correlations, single-cell localization, virtual network perturbation, and disease-model validation. However, the present study does not demonstrate that air pollutants induce CCL22 or that CCL22 mediates pollutant-associated AD. Exposure-resolved cohorts, controlled pollutant models, and target-specific functional experiments are required to test the proposed associations.

## Data Availability

The original contributions presented in the study are included in the article/Supplementary material, further inquiries can be directed to the corresponding author.
