# Supplementary material for: Integrative transcriptomic analysis identifies CCL22-associated immune signatures in air pollution-related atopic dermatitis
Source: Front Public Health. 2026 Sep 3;14:1906298. doi: 10.3389/fpubh.2026.1906298 (PMC13582549; doi:10.3389/fpubh.2026.1906298)
Supplement: Supplementary file 1 [file Data_Sheet_1.docx]

**Figure S1. Pollutant-specific ecological associations between air pollution and atopic dermatitis burden across country-year observations.**

(A–C) Country-year correlation analyses between selected air pollution indicators and AD burden metrics, including ASPR (A), ASIR (B), and ASDR (C). HAP and PM pollution showed inverse correlations with AD burden metrics, whereas NO₂ showed consistent positive correlations with ASPR, ASIR, and ASDR. Ozone showed weaker but statistically significant positive correlations with AD burden. Each dot represents one country-year observation. Blue curves indicate fitted trends. Spearman’s rho and P values are shown within each plot.

**Figure S2. Expression patterns and SHAP-based interpretation of the pollutant-related AD diagnostic signature.**

(A) Boxplots showing the expression levels of selected diagnostic signature genes, including **F2RL1, ICAM1, CCL5, IL18, CSF2, IL10, HRH4, IL17A,** and **IFNG**, between control and AD samples. Most genes did not show statistically significant differences in univariate comparisons.
(B) SHAP summary plot showing the distribution of SHAP values for the ten-gene diagnostic signature. Each point represents one sample, and color indicates the gene expression z-score.
(C) Bar plot showing the mean absolute SHAP values of individual signature genes, reflecting their relative contributions to the optimal machine learning model.
